# Study on the health risk of cyanuric acid in swimming pool water and its prevention and control measures

**DOI:** 10.3389/fpubh.2023.1294842

**Published:** 2024-01-08

**Authors:** Zhe Chen, Yi Su, Jian Chen, Zhu Li, Ting Wang

**Affiliations:** Shanghai Municipal Center for Disease Control and Prevention, Shanghai, China

**Keywords:** swimming pool, water quality testing, cyanuric acid, health risk, public health, review

## Abstract

Cyanuric acid is a widely used fine chemical intermediate that acts as a free chlorine buffer in swimming pool water, wherein it is often used as a stabilizer to maintain the germicidal efficacy of chlorinated disinfectants. However, it has also been associated with health risks. Herein, we introduced the sources and functions of cyanuric acid in swimming pool water, focusing on potential health risks associated with excessive concentration of the component and the current control standards worldwide. Also, the prevention and control measures were summarized in terms of physical chemistry, biodegradation, and ultraviolet radiation to provide a basis for the development of public health policies for swimming pool management.

## Introduction

Swimming pools are public bathing places where many people share the same water. Therefore, disinfecting the pool water is crucial to prevent the spread of diseases and inactivate microbial pathogens. Chlorine-containing disinfectant products are the most commonly used disinfectants in public swimming pools that inactivate various bacteria and viruses. However, since chlorine can be easily degraded by solar ultraviolet radiation, cyanuric acid is used in outdoor swimming pools to bind to chlorine for protection against ultraviolet degradation (1). Nonetheless, at high concentrations, cyanuric acid can lead to some health risks (2–4). Moreover, in the presence of cyanuric acid, chlorine is inactivated against intestinal pathogens, exposing swimmers to an increased risk of acute gastrointestinal diseases (5). Therefore, additional studies on the role of cyanuric acid in swimming pool water and the potential health risks and prevention and control measures that may result from its excessive concentration would help in ensuring public health safety in swimming pools.

## Application of cyanuric acid In swimming pool water

Products based on cyanuric acid have a history of use as disinfectants in swimming pools. As early as 1958, chlorinated cyanurates were used to disinfect swimming pool water in the United States (6). In 1974, Canelli discussed the chemical, bactericidal, toxic properties and the application of cyanuric acid and chlorinated cyanurates in swimming pools (7). In addition, chlorine in swimming pool water helps in the removal of bacteria, algae, dust, and dirt from the water, maintaining a clean environment for swimmers. However, cyanuric acid does not decompose easily or remove naturally from the water. Hence, at high concentrations, cyanuric acid aggregates in water over a period, reducing the disinfection effect of hypochlorous acid and posing a serious threat to human health. This early application of cyanuric acid is essential to maintain adequate chlorine levels consistently, providing a stable and effective disinfection environment in swimming pools. Thus, chlorine stabilization prolongs cyanuric acid’s lifespan in the water, ensuring a consistent and lasting disinfection effect (8). When the concentration of cyanuric acid reaches >200 mg/L in swimming pools, chlorination disinfection produces a “chlorine lock” effect, following which additional chlorine disinfectants in the swimming pool do not generate sufficient free chlorine. Therefore, since a high concentration inhibits the effect of chlorination disinfection, the concentration of cyanuric acid should be in a reasonable range (9) to maintain the water quality of the swimming pool. Unlike many other disinfectants, cyanuric acid protects chlorine from sunlight-mediated degradation, thus prolonging its effectiveness in sanitizing pool water (10). This stabilization effect facilitates a sustained and consistent disinfection process, making cyanuric acid a valuable component of long-term sanitation in swimming pools.

Melamine is highly soluble in water with a Log Pow value of −0.6 (11), and the solubilities of urea and cyanuric acid were determined in the temperature range of 300.75–369.45 K (12). Cyanuric acid has been found to correlate with several other water quality indexes in swimming pools. For example, in swimming pools, it is positively correlated with free residual chlorine concentration. In pool water, if the disinfectant exerts a bactericidal effect (99.999%) on *Escherichia coli, Streptococcus faecalis*, and *Staphylococcus*, more disinfectants should be added to water to achieve a high concentration of free chlorine; this, in turn prolongs the time required to kill the pathogens in the water (13). For example, at a pH 7.0 and temperature 25°C, 0.5 mg/L free active chlorine and 30 mg/L cyanuric acids need significantly longer time to inactivate 99.9% of the virus compared to free active nitrogen alone by a factor of 4.8–28.8 (14).

## Study on health risk and control standard of cyanuric acid in swimming pool water

### Health risks of cyanuric acid

Cyanuric acid is a by-product of the process of adding disinfectants to swimming pool water. Typically, the toxicity of cyanuric acid is low, and the toxicity in humans after oral or dermal exposure to cyanuric acid is negligible. Most people tend to excrete >98% of the ingested cyanuric acid through urine within 24 h, and does not significantly affect human metabolism (15). Therefore, cyanuric acid concentration levels in swimming pool water are employed to assess the volume of pool water ingested by swimmers and to monitor the health risks associated with swimming pools (16). Sinclair et al. (8) reported differences in the ability to metabolize cyanuric acid in various populations. The proportion of individuals with cyanuric acid excretion rates <80% within 24 h reached >25%, suggesting this swimmer’s pool water intake assessment index was not precise and may underestimate the intake level. Although the toxicity of cyanuric acid is not high, it may negatively affect human health. For example, cyanuric acid may irritate the eyes, skin, and respiratory system and damage the gastrointestinal tract or liver (17, 18). In addition, oral administration of cyanuric acid may seriously affect animal health. Considering the adverse effects of cyanuric acid on human health, the U.S. Environmental Protection Agency has classified cyanuric acid as one of the drinking water pollutants. The potential health risks associated with cyanuric acid stem from its ability to inhibit the efficiency of chlorine in disinfection. The excessive levels of cyanuric acid reduce chlorine activity, facilitating microbial proliferation in pool water. This compromised disinfection may increase the risk of waterborne illnesses due to inadequate elimination of bacteria, viruses, and other pathogens. Irrespective of ingestion or skin contact, direct exposure to high concentrations of cyanuric acid can lead to irritation, gastrointestinal distress, and in extreme cases, severe health effects.

In addition, when cyanuric acid and melamine are simultaneously present in the swimming pool water, the toxicity increases compared to cyanuric acid alone. This phenomenon may have an adverse effect on the urinary system of the human body, causing kidney and bladder stones and leading to kidney failure in severe cases (19). Occasionally, melamine is detected in the swimming pool water due to various factors, primarily related to its use in pool-related products or accidental contamination, especially in less regulated regions where it might be utilized in pool construction or maintenance (10). Several studies have highlighted the presence of melamine in food and its association with kidney toxicity, especially in cases of co-exposure to cyanuric acid and phthalates, emphasizing the risk of early kidney impairment. Dorne et al. (20) and Liu et al. (21) examined the interactions between these compounds and their effects on kidney health. The mechanism of melamine toxicity involves the formation of crystals with cyanuric acid in renal tubules, potentially leading to acute kidney failure. The co-exposure of cyanuric acid and melamine in animals and humans exhibited increased toxicity compared to individual exposure. Moreover, Xu et al. (22) underscored the complex toxic effects arising from co-exposure to melamine and cyanuric acid, showing cytotoxic and genotoxic effects in human kidney cells, which in turn suggested the formation of melamine-cyanuric acid complex.

Furthermore, cyanuric acid is implicated in pathological changes in other organs, including the spleen (23), liver (24), uterus (25), and humoral immune function (26). A recent study demonstrated that cyanuric acid inhibits presynaptic neurotransmission in a dose-dependent manner by suppressing the frequency of excitatory postsynaptic currents in the hippocampal slices (27). This phenomenon might be attributed to changes in residual calcium concentration at presynaptic terminals, as demonstrated by enhanced paired pulses (27). Interestingly, exposure to 10, 20, and 40 mg/kg cyanuric acid for four weeks severely damages thyroid hormone homeostasis, leading to anxiety-and depression-like behaviors (28). Therefore, it can be deduced that high concentrations of cyanuric acid induce neurotoxicity in various products (29). Melamine and cyanuric acid exhibited low toxicity individually but high toxicological effects upon co-exposure. Also, co-exposure leads to significant oxidative stress and damage to placental development, resulting in increased apoptosis and decreased fetal cell proliferation (30). The co-exposure severely impairs neuronal and synaptic functions, reducing the amplitude, decay time, and frequency of miniature excitatory postsynaptic currents (mEPSCs) without affecting miniature inhibitory postsynaptic currents (mIPSCs) (31). Prenatal exposure to cyanuric acid elevates alkaline phosphatase levels in the maternal placenta and fetal brains (32).

### Limited standard of cyanuric acid in swimming pool water

In order to regulate the concentration of cyanuric acid in swimming pools, all countries have clear standards, and most have set a control range of 10–200 mg/L in the swimming pool water (13). For example, the American standard for cyanuric acid in swimming pool water is at least 10 mg/L, preferably 30–50 mg/L, but not >150 mg/L. The Australian standard requires that the maximum concentration of cyanuric acid should not exceed 100 mg/L when used as a chlorine stabilizer. Also, cyanuric acid should not be used in indoor swimming pools and public spas. The British standards require that the maximum cyanuric acid concentration in a swimming pool should be <200 mg/L and optimally between 50 and 100 mg/L. The World Health Organization (WHO) has recommended the standard of cyanuric acid concentration in swimming pool water, according to which cyanuric acid should not be detected in indoor swimming pools, while the concentration in outdoor swimming pools should be <100 mg/L. The International Swimming Pool Foundation (NSPF) recommended maintaining the cyanuric acid concentration in swimming pool water between 10 and 100 mg/L. In China, certain standards are mainly related to swimming pool water quality indicators, such as “hygienic standard for swimming places” (GB 9667–1996) (33), “water quality standards for swimming pools” (CJ 244–2007; CJ/T 244–2016) (34, 35), and “sanitary indicators and limit requirements for public places” (GB 37488) (36). Among these, GB 9667–1996 does not have clear regulations on the concentration of cyanuric acid; CJ 244–2007 stipulates that the concentration of cyanuric acid in swimming pool water should be controlled <150 mg/L; CJ/T 244–2016 subdivides the cyanuric acid concentration standard in swimming pool water, stipulating that the cyanuric acid concentration in an outdoor swimming pool should be <30 mg/L, while that in an outdoor swimming pool should be <100 mg/L; GB 37488–2019 stipulates that the cyanuric acid limit of swimming pool water should be 50 mg/L.

### Detection of cyanuric acid in the swimming pool

The concentration of cyanuric acid in pool water is determined using various methods: liquid chromatography-mass spectrometry (LC–MS) (37), ultra-performance liquid chromatography-mass spectrometry (UPLC-MS) (11), and gas chromatography–mass spectrometry (GC–MS) (38). These techniques utilize chromatography to separate the compounds and mass spectrometry to identify and quantify cyanuric acid based on its unique molecular characteristics. LC–MS and UPLC-MS offer high sensitivity and accuracy in detecting cyanuric acid, while GC–MS, although less commonly used, is an alternative method for its identification through vapor-phase separation. These methods measure cyanuric acid levels in pool water, ensuring compliance with safety standards and appropriate disinfection.

Currently, only a few studies have detected the levels of cyanuric acid in swimming pool water, while many have employed the cyanuric acid concentration index to determine the quality of swimming pool water. Xiuying et al. (39) determined that the cyanuric acid levels in swimming pool water in the Dongcheng District of Beijing between April and August 2018 were 23.0 mg/L, with a qualified rate of 70.6%. Factors such as the type of establishment operation, structural design, type of shower equipment, and the way of disinfection were shown to have a significant impact on the qualified rate of cyanuric acid. Among these, the qualification rate of the school swimming pool was the highest, followed by hotels and restaurants, while social profit swimming pools had the lowest rates. The qualified rates of cyanuric acid in swimming pools with mandatory showers and automatic machine dosing were significantly higher compared to pools without mandatory showers and manual dosing. The swimming pool temperature and urea showed a positive correlation with cyanuric acid content. Liang et al. examined the cyanuric acid content in water of 21 swimming pools in Kunshan City (40) and found that the concentration range was 2–79 mg/L, with a median of 20.0 mg/L. Strikingly, the cyanuric acid qualifying rate was highest in open-air pools with cyanuric acid concentrations up to 10 mg/L, and the qualified rate was higher in indoor swimming pools with a window than in those without a window. The residual chlorine in water between 0.2 and 0.6 mg/L eliminated the pathogenic bacteria, such as *Escherichia coli* and *Staphylococcus aureus*. Based on the water quality standard for swimming pools (CJ/T 244–2016), Pei et al. monitored and analyzed swimming pools in Changzhou from 2015 to 2016. The study showed that the qualified rate of cyanuric acid in outdoor swimming pools was 100%, while that in indoor swimming pools was 77% (41). Based on the standard CJ 244–2016, Haixia et al. monitored the water quality of swimming pools in Chaoyang District, Beijing, in 2017 and found that the qualified rate of cyanuric acid in swimming pool water was 73.96%; the change in the qualified rate of cyanuric acid was similar in different months (42).

## Prevention and control measures of cyanuric acid health risk in swimming pool water

The requirements for adding a cyanuric acid disinfectant to the pool water in terms of concentration are based on the water quality standards of swimming pools worldwide. Thus, cyanuric acid can be used for disinfection without any obvious damage to human health. The common methods to remove excess cyanuric acid include physicochemical, biodegradation, and ultraviolet irradiation.

### Physicochemical method

The physicochemical method includes the removal of high concentrations of cyanuric acid (> 50 ppm), which is achieved by partially or completely emptying the pool and replacing the old with fresh water. The method can be subdivided into water supplementation and coagulation sedimentation filtration. The water replenishment method is effectuated by continuously replenishing fresh water in the swimming pool. The major disadvantages of this method are cost and long duration. If water replenishment is carried out for large swimming pools, the replenishment volume should reach 25–50% of the total volume, which increases the operating cost of the swimming pool and causes a significant waste of water. Moreover, this method is not suitable for drought periods or areas (21) with scarce water resources. The coagulation sedimentation filtration method is based on a chemical reaction between melamine and cyanuric acid to produce a new compound (melamine cyanurate), which is precipitated and filtered to remove cyanuric acid from the pool water. Also, flocculants can be added to accelerate the precipitation effect of the compound (4). However, this method has some limitations, i.e., after the chemical reaction between melamine and cyanuric acid, the color of swimming water becomes green within 1–2 weeks and is usually negatively viewed by many swimmers (43). Somella et al. proposed a novel approach to remove cyanuric acid from swimming pools without the water turning green. The basic principle of this method is to add a residual chlorine neutralizer (such as sodium sulfite) to the pool water to decrease the chlorine content and then produce a precipitation compound through the chemical reaction between melamine and cyanuric acid. Subsequently, the precipitates are discharged through the filtration system of the swimming pool to remove cyanuric acid from the pool water without turning its green.

### Biodegradation

The principle of biodegradation involves the degradation of cyanuric acid by adding hydrolytic enzyme active components to the pool water. In some studies, whole immobilized cells (44) expressing endogenous cyanuric acid hydrolase (33) or isolated hydrolase were utilized (45). Some studies (46) reported that N-methylisocyanuric acid is an excellent substrate for cyanuric acid hydrolase at a kcat of 3.1 ± 0.3 s^−1^ and a Km of 71 ± 10 μM by testing the reaction of dozens of compounds with cyanuric acid. In addition, at a kcat/Km of 4.4 × 104 s^−1^ M^−1^, N-methylisocyanuric acid and cyanuric acid measurements were of the same magnitude. However, N, N-dimethyl and N, N, N-trimethyl isocyanuric acid had no obvious reactivity with cyanuric acid hydrolase, indicating high substrate differentiation. Thus, introducing whole cells into the pool water is less efficient, and the whole treatment may take up to 2 weeks while applying cell-free cyanuric acid to the pool is more straightforward and effective. Showell et al. proposed a comprehensive approach for the removal of cyanuric acid using a combination of reagents, such as *Bacillus*, *Lactobacillus*, *Pseudomonas*, carbon sources (for example, carbohydrates, proteins, and polysaccharides), and inorganic minerals (such as disodium hydrogen phosphate, dipotassium hydrogen phosphate, and sodium chloride) (47); the combination of these reagents can remove cyanuric acid from water without harboring pathogenic microorganisms. In addition, *Moorella thermoacetica* (ATCC 39073) produce hydrolase capable of degrading cyanuric acid in water. This hydrolase has heat-resistant characteristics and great potential to be used in the degradation of cyanuric acid in swimming pool water. Moreover, it is difficult for the hydrolytic enzymes produced by this bacterium to remain active during the oxidation of the disinfectant, thereby affecting their effectiveness in removing cyanuric acid (48). However, aminopropyltriethoxysilane (APTES) can be used to reduce the extent to which disinfectants affect the activity of hydrolases, thus improving the removal of cyanuric acid from pool water (49). Presently, only a few enzyme products can be used for industrial applications because of the high capacity of common swimming pools. This phenomenon requires a high amount of cyanuric acid, and the potential denaturation of cyanuric acid is effectuated by residual free chlorine. Engineered hydrolases with C46A mutations improve hypochlorite resistance but are insufficient for use in swimming pools due to hypochlorite sensitivity, enzyme productivity, and thermal stability (50). In a recent study (2), Guo et al. identified a previously unknown cyanuric acid hydrolase from *Pseudolabs* sp. Root1462 (CAH-PR) by mining; the potential bioinformatics of cyanuric acid hydrolase genes were analyzed from public databases. The enzyme is suitable for industrial application in cell-free form based on its favorable enzymatic and physical properties combined with high-yield expression in aerobic cell culture. The kinetic parameters and model structure are similar to those of known cyanuric acid hydrolases; however, this new enzyme exhibited excellent thermal and storage stability.

### Ultraviolet irradiation method

Previous studies have shown that UV radiation decomposes cyanuric acid, thus reducing the content of cyanuric acid in swimming pools (51). According to the literature, cyanuric acid content is lower in the water of outdoor swimming pools or where UV disinfection is applied to disinfect the pool. In swimming pools without ultraviolet radiation, stabilizing the residual chlorine content in the pool water is challenging, and it might pollute the water quality compared to swimming places with UV irradiation. Nonetheless, it should be pointed out that ultraviolet radiation cannot fundamentally resolve the issue of cyanuric acid overload. In order to control the cyanuric acid content of swimming pools, it is necessary to control disinfectant input. Moreover, the amount of cyanuric acid disinfectant should be determined according to whether the swimming pool is outdoors and whether it has ultraviolet disinfection equipment. For outdoor pools or those with UV disinfection equipment, the disinfectant input can be increased, and a combination of disinfectants or disinfection methods can be applied. This approach reduces the dependence on cyanuric acid-based disinfectants and the potential health risks posed by cyanuric acid accumulation.

## Conclusion

Cyanuric acid can promote the role of chlorinated disinfectants in the swimming pool’s disinfection process; however, the accumulation of cyanuric acid in swimming pools may lead to new health risks. The common approaches to control cyanuric acid concentration in swimming pools include physicochemical, biodegradation, and ultraviolet radiation methods. However, these methods vary in cost, efficacy, and application. Therefore, cyanuric acid health risk prevention and control measures should be selected cautiously according to the specific type of swimming pool, water quality requirements, and cost efficiency to ensure maximum disinfection effect of cyanuric acid while avoiding potential health risks caused by excessive disinfectants. In order to control the concentration of cyanuric acid in swimming pool water, additional studies are required to establish the standards suitable for Chinese and international swimming pool water. This would aid in comparing the effects of different methods to control the concentration of cyanuric acid in swimming pools and determine the appropriate conditions of various methods, thus improving their effectiveness.

## Author contributions

ZC: Writing – original draft. YS: Writing – original draft. JC: Funding acquisition, Supervision, Writing – review & editing. ZL: Funding acquisition, Supervision, Writing – review & editing. TW: Writing – original draft.
